# Efficacy and safety analysis of chemotherapy combined with immunotherapy compared with standard chemotherapy for advanced biliary tract malignant tumors

**DOI:** 10.1002/cam4.6209

**Published:** 2023-07-01

**Authors:** Zhengfeng Zhang, Dazhen Wang, Liu Yang, Lu Zhao, Lei Yang, Jianji Zhang, Changjie Lou

**Affiliations:** ^1^ Department of Gastroenterology Harbin Medical University Cancer Hospital Harbin China; ^2^ Mianyang Central Hospital Mianyang China

**Keywords:** advanced biliary tract cancer, chemotherapy, efficacy, immunotherapy, safety

## Abstract

**Objective:**

The treatment of biliary tract (BTC) cancer remains relatively limited, especially in the setting of advanced BTC. Immune checkpoint inhibitors (ICIs) have shown some effects in a variety of solid tumors, but their efficacy and safety in patients with advanced BTC are still elusive, which require in‐depth analysis.

**Methods:**

The clinical information of 129 patients diagnosed with advanced BTC between 2018 and 2021 were retrospectively reviewed. All patients were treated with chemotherapy, while a portion of them (64 patients) were treated with ICIs, the other 64 patients were not. Therefore, we divided the patients into two groups, SC (standard chemotherapy) and CI (chemotherapy in combined with immunotherapy), then we analyzed the benefit of adding ICIs according to efficacy, adverse events, progression‐free survival (PFS), progressive disease (PD), and the influence of various factors and effectiveness.

**Results:**

The mean PFS was 9.67 months for CI group and 6.83 months for SC group. The PFS was prolonged by 2.84 months with ICI addition, and the difference was statistically significant (*t* = 3.114, 95% CI: 1.06–4.74, *p* < 0.001). The objective response rate (ORR) was 32.81% (21/64) for the CI group versus 10.77% (7/65) for the SC group, and the disease control rate (DCR) was 79.69% (51/64) versus 67.69% (44/65), respectively. Regression analysis showed that factors such as changes in CA19‐9, the level of PD‐L1 expression, tobacco and alcohol, and the neutrophil–lymphocyte (NLR) ratio all influenced PFS (*p* < 0.05 for all these factors). For the treatment‐related adverse events (TRAEs), the highest grade 3–4 adverse effects were thrombocytopenia in 7.75% (10/129) and neutropenia in 3.1% (4/129), immune‐related adverse events (irAEs) occurred in 32.8% (21/64), and all were grade 1–2.

**Conclusions:**

Our results showed that ICIs combined with chemotherapy exhibited good antitumor activity with acceptable safety and could be recommended as first‐line treatment for patients with advanced BTC.

## Abstract 2

ICIs was effective in prolonging PFS in patients with advanced BTC; in addition, histological grade, PD‐L1 expression, NLR ratio, optimal efficacy evaluation, prior tobacco and alcohol history, TMB were associated with prognosis; and the decreased level CA19‐9 and NLR ratio were predictors of efficacy and prognosis.

## 1 INTRODUCTION

Biliary tract cancer (BTC) accounts for approximately 3% of digestive malignancies and is a rare tumor with high malignancy, rapid progression, and extremely poor prognosis,^1^, ^2^ less than 20% of patients with advanced BTC have the opportunity for surgery^3^ and the 5‐year postoperative survival rate is approximately 7%–20%.^4^, ^5^ Once a patient is diagnosed with advanced BTC, there are fewer effective therapies available, primarily gemcitabine‐based chemotherapy, but it may not significantly improve patient survival.^6^ Targeted therapies including targets such as neurotrophin receptor kinase (NTRK), fibroblast growth factor receptor 2 (FGFR2), and isocitrate dehydrogenase 1 (IDH1) have made considerable success, unfortunately only 5% of patients are eligible to be applied.^7^, ^8^

In recent years, immunotherapy has made some progress in the treatment of multiple solid tumors, it has also shown certain potential in BTC, and a number of related clinical trials are being undertaken. The mOS and mPFS for patients with advanced BTC treated with pembrolizumab in KEYNOTE‐028 clinical trial were 5.7 months (3.1–9.8) and 1.8 months (1.4–3.1) respectively, with an objective response rate (ORR) value of 13%.^9^ And the KEYNOTE‐158 clinical trial found that advanced BTC patients with positive expression of programmed death receptor 1 (PD‐L1) had a better prognosis than those with negative expression level; Patients with mismatch repair deficiency (dMMR) or high microsatellite instability (MSI‐H) also had a high ORR of 40.9%, and even 17% of patients achieved partial remission (PR) that lasted for a longer period.^2^, ^10^, ^11^ Although these aforementioned clinical trials are all phase I/II, it can still be found that patients with advanced BTC may benefit from single‐agent immunotherapy, especially those with positive PD‐L1 and dMMR/MSI‐H expression. Although pembrolizumab is included in the guidelines of the Chinese Society of Clinical Oncology (CSCO) for the disease in 2021, it is mainly aimed at the subsequent treatment of patients with advanced BTC after first‐line treatment failure. However, in several phases I/II clinical trials using distinct immune checkpoint inhibitors (ICIs) for subsequent treatments in patients with advanced BTC after first‐line treatment failure, ICIs did not demonstrate superior efficacy,^12^ it is therefore recommended to use two or more ICIs together. In a phase II clinical trial (NCT02923934), researchers used nivolumab and ipilimumab in combination, and found that the ORR was only 23%, which showed no obvious benefit compared with using single ICI, but the cost of treatment increased accordingly.^13^

Given the fact that ICI combined with chemotherapy has shown good efficacy in first‐line treatment of various solid tumors, but there is still a lack of evidence to support the combination in first‐line treatment of patients with advanced BTC, considering only one clinical trial III has achieved positive results. Therefore, we carried out this retrospective study to explore the practical value of ICI in the first‐line treatment of patients with advanced BTC, the primary goal is to provide clinical evidences to support the use of ICI in combination with chemotherapy as first‐line therapy for patients with advanced BTC.

## 2 DATA AND METHOD

### 2.1 The inclusion and exclusion criterion

Our study included patients with advanced BTC who were treated at Harbin Medical University Cancer Hospital from 2018 to 2021. The inclusion criteria were as follows: (1) pathologically diagnosis as BTC, including cholangiocarcinoma (CCA) and gallbladder cancer (GCA); (2) patients with advanced BTC with measurable target lesions; (3) receiving at least two single regimen cycles, and had at least one efficacy evaluation; (4) Eastern Cooperative Oncology Group (ECOG) Score: 0–2 points. The exclusion criteria were as follows: (1) had additional active tumors; (2) had no measurable target lesion; (3) missing or incomplete clinical data; and (4) had no complete follow‐up information.

### 2.2 Therapeutic regimen

Chemoimmunotherapy group (CI; experimental group): three therapeutic regimens of the first line (GP, GS: Gemcitabine+S‐1 and GEMOX) + ICIs; standard chemotherapy group (SC; control group): Three therapeutic regimens of the first line (GP, GS, and GEMOX) with no additional ICIs treatment. The treatment plan is based on the Chinese Society of Clinical Oncology(CSCO) guidelines.

### 2.3 Research method

#### 2.3.1 Clinical information

The clinical information includes patient's name, gender, age, height, weight, history of tobacco and alcohol, the location of tumor, degree of histological differentiation, with additional ICIs treatment or not, different types of ICIs, ECOG score, the time to the first definite diagnosis, have been treated for surgery or not, whether adjuvant chemotherapy, combined with radiotherapy or not, combined with radiofrequency ablation treatment or not, combined with hepatic arterial perfusion chemotherapy, results of the hematological examination, imaging examination findings, genetic test results, efficacy evaluation, the time to disease progression, the causes of disease progression, treatment‐related adverse events (TRAEs), survival status up to the last follow‐up visit, time of death, and cause of death, etc.

Follow‐up data included visits to hospital case data, readmissions, and telephone contacts. The most recent follow‐up visit took place in July 2022. Progression‐free survival (PFS) was defined as the time from the start of the first course of treatment until the patients' progression or death, OS (not yet reached OS) is the time from the start of the first course of treatment until death (for any reason).

Ethical statement: The retrospective study was approved by the Ethics Committee of Harbin Medical University Cancer Hospital prior to conducting the study (approval number is: KY2022‐14).

#### 2.3.2 Response evaluation criteria

The effectiveness of both groups was assessed using the Response Evaluation Criteria in solid tumors (RECIST1.1), which can be further divided into complete response (CR), partial response (PR), stable disease (SD), and progressive disease (PD). If the CI group has a negative progression after evaluation, it will be reassessed according to the modified RECIST1.1 (iRECIST1.1). We classified TRAEs according to Toxicity Criteria version 5.0. Disease control rate (DCR) is defined as the proportion of PD cases post‐medication, and ORR is defined as the percentage of patients with CRs and PRs.

#### 2.3.3 Statistical methodology

SPSS26.0 software (SPSS 26.0 software—IBM Corp. Released 2019. IBM SPSS Statistics for Windows, Version 26.0. IBM Corp) was used for statistical analysis. Data from continuous measurements that followed a normal distribution were reported as mean ± SD, and data from normally distributed and homogenous variance measurement were compared by one‐way ANOVA with post hoc pairwise comparisons using the LSD method. And data that did not follow a normal distribution and having unequal variance were analyzed by the Kruskal–Wallis test. The chi‐square test was used for the comparison of count data between groups, expressed by usage rate or composition ratio. If the bivariate was normally distributed, the Pearson's linear correlation was used to analyzed the data. The Spearman rank correlation was used to analyze the data of non‐normal distribution. Multivariable analysis was conducted using logistic regression analysis. *p* value less than 0.05 was considered to be statistically significant. Survival curves were plotted using the Kaplan–Meier method and the survival rate was compared by a log‐rank test.

## 3 RESULTS

### 3.1 Analysis of clinical data in both arms

**FIGURE 1:**
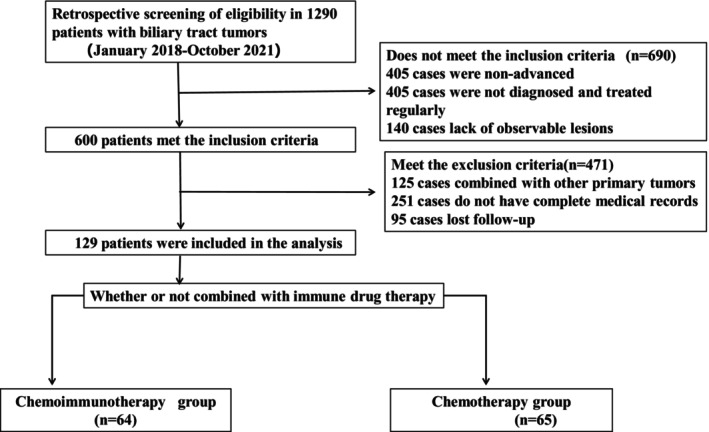
The flow chart of this study.

**FIGURE 2:**
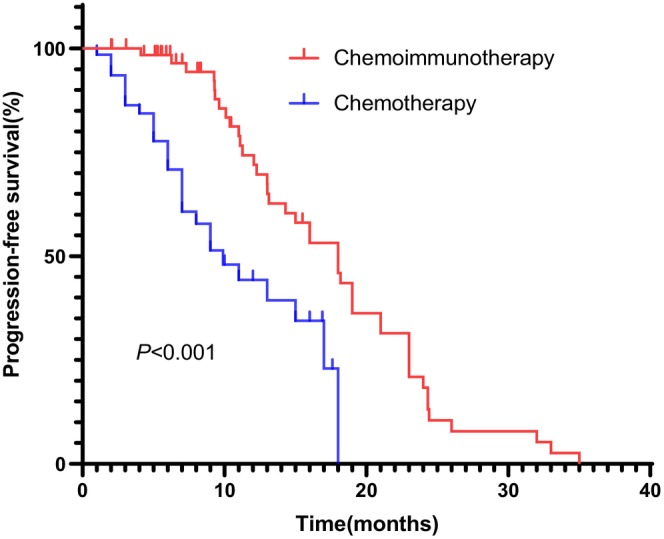
Kaplan–Meier curves of progression‐free survival between standard chemotherapy (SC) and chemoimmunotherapy (CI) groups.

The current study enrolled 129 patients with advanced BTC receiving first‐line treatment (Figure 1 and Table 1). Patients were divided into two groups, SC and CI, according to whether additional ICIs were treated. The CI group referred to the chemoimmunotherapy arm with 64 patients; the SC group referred to the chemotherapy arm with 65 patients. All patients had ECOG scores ranging from 0 to 1. There were no statistically significant differences in age (*p* = 0.971), sex (*p* = 0.811), height (*p* = 0.376), weight (*p* = 0.454), tumor location (*p* = 0.881), and so forth between the SC and CI groups. The observed PFS in the CI group (9.67 ± 5.42) was significantly higher than that in the SC group (6.83 ± 5.16) (*p* < 0.0001) (Figure 2).

**TABLE 1:** Comparison of disease characteristics in patients with advanced biliary tract tumors.

Variables	CI group (*n* = 64)	SC group (*n* = 65)	*p*‐value
Use ICIs	Yes	No	**<0.001**
Gender			0.087
Male	37 (57.8)	44 (67.7)	
Female	27 (42.2)	21 (32.3)	
Age (mean ± SD)	57.12 ± 8.99	62.38 ± 10.19	0.327
<60	34 (52.3)	25(38.5)	
≥60	30 (46.2)	40(61.5)	
Smoking or drinking			**0.015**
Yes	11 (17.2)	16 (24.6)	
No	53 (82.8)	49 (75.4)	
Tumor location			0.881
ICC	22 (34.4)	19 (29.2)	
HCCA	5 (7.8)	15 (23.1)	
DCCA	11 (17.2)	2 (3.1)	
GCA	12 (18.8)	13 (20.0)	
Unknown	14 (21.9)	16 (24.6)	
Histologic differentiation			**0.029**
Well	3 (4.7)	3 (4.6)	
Moderately	22 (34.4)	15 (23.1)	
Poorly	19 (29.7)	17 (26.2)	
Unknown	20 (31.1)	29 (44.6)	
Number of recurrent organs			**0.006**
Single	23 (36.0)	14 (21.5)	
Multiple	41 (64.0)	51 (78.5)	
Radical surgery			0.543
Yes	34 (53.1)	24 (36.9)	
No	30 (46.9)	41 (63.1)	
Adjuvant chemotherapy			0.287
Yes	14 (21.9)	14 (21.5)	
No	50 (78.1)	51 (78.5)	
Radiation therapy			0.0397
Yes	10 (15.6)	7 (10.8)	
No	54 (84.4)	58 (89.2)	
Viral hepatitis			0.953
Yes	7 (10.9)	8 (12.3)	
No	43 (67.2)	40 (61.5)	
Unknown	14 (21.9)	17 (26.2)	
Types of immune drugs		—	0.709
Camrelizumab	15 (23.4)	—	
Pembrolizumab	9 (14.1)	—	
Toripalimab	20 (31.3)	—	
Sintilimab	11 (17.2)	—	
Others	9 (14.0)	—	
Evaluation of curative effect			**<0.001**
CR	2 (3.1)	0	
PR	19 (29.7)	7 (10.8)	
SD	30 (46.9)	37 (56.9)	
PD	13 (20.3)	21 (32.3)	
NLR			**0.003**
<2.30	28 (43.8)	45 (69.2)	
≥2.30	36 (56.2)	20 (30.8)	
PD‐L1 (CPS score %)		—	**0.0089**
≥1%	18 (28.1)	—	
<1%	9 (14.1)	—	
Unknown	37 (57.8)	—	
Increase in bilirubin			0.172
Yes	29 (45.3)	21 (32.3)	
No	35 (54.7)	44 (67.7)	
Increase in transaminase			**0.002**
Yes	44 (68.8)	26 (40.0)	
No	20 (31.2)	39 (60.0)	
Increase in tumor markers			0.593
Yes	49 (76.6)	52 (80.0)	
No	15 (23.4)	13 (20.0)	
CA19‐9 decrease			**0.0368**
Yes	33 (51.6)	27 (41.5)	
No	31 (48.4)	38 (58.5)	
Microsatellite state		—	0.3722
MSI‐L	5 (7.8)	—	
MSS	7 (10.9)	—	
MSI‐H	3 (4.7)	—	
Unknown	49 (76.6)	—	
TMB value (muts/Mb)		—	0.077
≥10	11 (17.2)	—	
<10	13 (20.3)	—	
Unknown	40 (62.5)		
ECOG			**0.015**
0	38 (59.4)	35 (53.8)	
1	26 (40.6)	30 (46.2)	

Abbreviations: CPS, combined positive score; ECOG, Eastern Cooperative Oncology Group; GCA, gallbladder carcinoma; HCCA, hila cholangiocarcinoma; DCCA, distal cholangiocarcinoma; ICCA, intrahepatic cholangiocarcinoma; MSI‐H, Microsatellite instability‐high; MSI‐L, Microsatellite instability‐low; MSS, Microsatellite stability; NLR, neutrophil‐lymphocyte ratio; PD, progressive disease; PD‐L1, programmed cell death‐Ligand 1; PR, partial response; SD, stable disease; TMB, tumor mutation burden.

*p* value < 0.05 are indicated in bold.

### 3.2 Efficacy analysis

**FIGURE 3:**
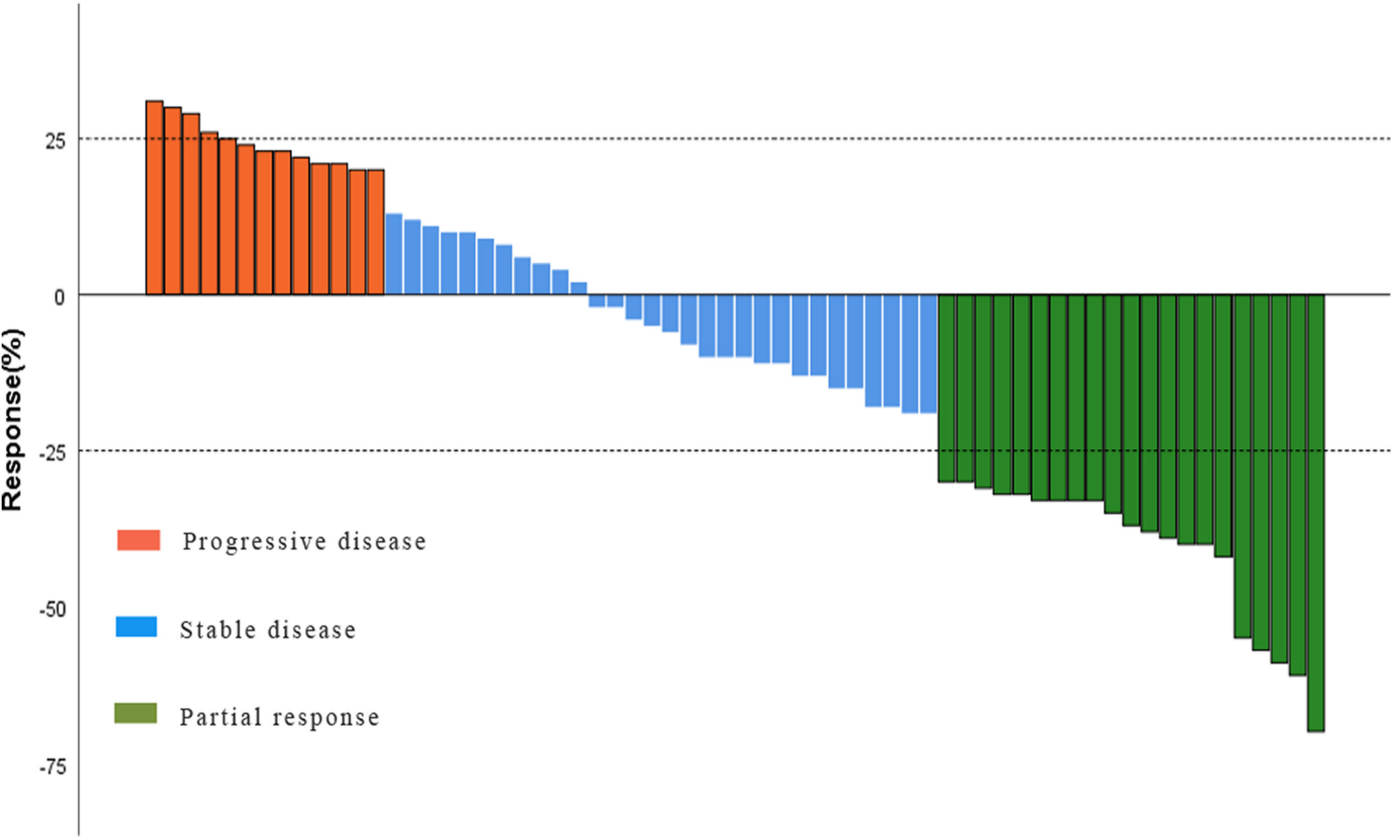
Maximum percentage reduction of target lesions from baseline in the CI arm (*n* = 64).

In the CI arm, all 64 patients received at least two treatments on a regular basis and had at least one efficacy assessment. 3.1% (2/64) of patients who achieved CR were CCA; 29.7% (19/64) of patients achieved PR, of which 28.1% (18/64) patients had CCA and 1.6% (1/64) patient had GCA; The percentage of patients achieving SD was 46.9% (30/64), of which 25 39.1% (25/64) patients had CCA and 7.8% (5/64) had GCA. Overall, 20.3% (13/64) of patients achieved PD, of which 10.9% (7/64) patients had CCA and 9.4% (6/64) had GCA. The ORR was 32.8% (21/64), and the DCR was 79.7% (51/64) (Figure 3).

In the SC arm, the mean treatment cycle was 5 ± 2 cycles, and all 65 patients underwent at least one assessment. The proportion of patients achieved CR was zero; the proportion achieved PR was 10.8% (7/65), and 9.2% (6/65) with CCA, and 1.6% (1/65) with GCA; the proportion achieved SD were 56.9% (37/65), of which 46.2% (30/65) were CCA and 10.7% (7/65) were GCA. The proportion of patients achieved PD were 32.3% (21/65), including 24.6% (6/65) with CCA and 7.7% (5/65) with GCA. The ORR was 10.8% (7/65), and the DCR was 67.7% (44/65).

Statistical analysis revealed that PFS in both arms did not correlate with age, gender, tumor location, history of hepatitis, whether surgical treatment was provided, site of tumor metastasis, different chemotherapy regimens, bilirubin level, and tumor mutation burden (TMB) (all *p*>0.05). However, the factors including history of tobacco and alcohol usage, decreased CA19‐9, neutrophil–lymphocyte (NLR) ratio <2.30, previously received chemotherapy and radiation for hepatic artery embolization, degree of histologic differentiation, exhibits statistical significant correlation with PFS (all *p* value<0.05). There was also a significant statistical correlation between PD‐L1 expression, MSI status, and PFS in the CI group.

By the time of the last follow‐up time (2021‐12‐03), among the 64 patients in the CI group, 27 (42.2%) had progressed to PD, including 20 (31.3%) of CCA and 7 (10.9%) of GCA. Six patients (9.4%) received radiation therapy during or after the combination therapy, including five (7.8%) CCA and one (1.6%) GCA. Although 13 patients (20.3%) did not progress to PD, they discontinued immunotherapy, including 10 (15.6%) CCA and three (4.7%) CGA. A total of 24 patients (37.5%) did not progress to PD and continued to receive ICI, including 22 (34.4%) patients with CCA and two (3.1%) with CGA. Of the 65 patients in the SC group, 51 (78.5%) progressed to PD, with 39 (60%) to CCA and 12 to GCA (18.5%). Seven patients (10.8%) received radiotherapy during or after combination therapy, including six (9.2%) CCA and one (1.6%) GCA. Univariate regression analysis was performed between various factors and PFS in the CI arm, it is found that there are statistical differences between PFS and factors including the decrease in CA19‐9 following treatment, the best assessment of efficacy was either CR or PR, NLR <2.30, no smoking or alcohol consumption, treatment is combined with hepatic artery embolization chemotherapy, or combined with radiation therapy, the PD‐L1 gene was found to be positively expressed, MSI‐H, with a high degree of histological differentiation (all *p* < 0.05). Then multivariate regression analysis was performed on the factors with *p <* 0.05, the results showed that NLR <2.30 (*p <* 0.001), PD‐L1 positive expression (*p* = 0.043), high degree of histological differentiation (*p* = 0.031), MSI‐H (*p* = 0.046) are the independent essential factors affecting PFS (Table 2).

**TABLE 2:** Univariate and multivariate analysis of progression‐free survival in combined treatment patients with advanced biliary tract malignant tumors.

Variables	Univariate analysis			Multivariate analysis		
	HR	95% CI	*p*‐value	HR	95% CI	*p*‐value
Primary site: intrahepatic	0.390	−3.111 to −3.890	0.825			
Well differentiation degree	1.368	0.278–2.457	**0.015**	1.473	0.695–2.251	0.031
NLR <2.30	−6.393	−8.607 to −4.178	**<0.001**	−4.349	−6.400 to −2.297	<0.001
Viral hepatitis	−1.115	−2.741–0.512	0.176			
PD‐L1 CPS score ≥1	2.227	0.545–3.909	**0.010**	0.825	0.242–2.072	0.043
Transarterial Chemoembolization	8.505	3.288–13.722	**0.002**	3.773	−0.322 to 7.868	0.070
Male	−0.216	−2.982 to 2.551	0.877			
Age ≤60	1.32	−1.399 to 4.038	0.336			
Smoking or Drinking	1.155	0.695–2.404	**0.012**	−1.416	−4.605 to 1.232	0.288
TMB >10 mut/Mb	5.718	−1.836 to 8.599	0.089			
Radiotherapy	6.047	2.610–9.484	**0.001**	1.202	−1.684 to 4.089	0.407
BMI <23.0	0.063	−0.383 to 0.508	0.780			
ALB ≥40 g/L	2.145	−0.536 to 4.825	0.115			
Transaminase decreased	2.356	−0.531–5.243	0.108			
CA19‐9 decreased	2.324	0.350 to 4.999	**0.047**	1.393	−0.641 to 3.427	0.175
MSI‐H	1.377	0.247–2.507	**0.018**	0.609	0.243–1.460	0.046
CR and PR	2.397	0.445–4.348	**0.017**	−0.518	−0.129 to 1.093	0.522
Types of immune drugs	0.734	−0.275 to 1.742	0.151			
ECOG = 0	0.484	0.281–0. 832	**0.0451**	1.701	8.889–3.213	0.126
Radiofrequency ablation	1.537	−9.383 to 6.308	0.697			
Surgery	1.767	−2.064 to 3.404	0.626			

*p* value < 0.05 are indicated in bold.

### 3.3 Treatment‐related adverse events (TRAEs)

Overall, the incidence of TRAEs in CI and SC group patients was 70.3% (45/64) versus 57.0% (37/65) respectively. In the CI arm, 54.7% (35/64) and 15.6% (10/64) of TRAEs were grade 1–2 and 3–4. The incidence of grade 1–2 and 3–4 TRAEs in the SC arm was 46.2% (30/65) and 10.8% (7/65). The highest incidence of TRAEs were decreased serum albumin (46.5%), increased bilirubin (29.5%), transaminase (27.1%), and neutropenia (22.5%). After infusion of human blood albumin, patients with decreased in serum albumin up to grade 3–4 returned to normal, and all patients with significantly elevated bilirubin and transaminase index underwent a significant remission after symptomatic therapy such as transcatheter arterial chemoembolization or following biliary stenting, and the presence of neutropenia during treatment was restored to the normal range following treatment with the injection of recombinant human granulocyte colony‐stimulating factor. All grade 3–4 TRAEs that occurred were controlled after the patient received symptomatic medications, and did not affect subsequent treatment.

The incidence of immune‐related adverse events (iRAEs) in the CI group was 37.5% (22/64), and all were grade 1–2. The highest incidence of iRAEs mainly included 13 (20.3%) cases of immune capillary, two (3.1%) cases of immune gastroenteritis, two (3.1%) cases of immune hyperthyroidism, one (1.6%) case of immune pneumonia, two (3.1%) cases of epidemic hypothyroidism, and two (3.1%) cases of immune fever. Thirteen patients with immune capillaries had mild symptoms, reported good tolerance, and continued use of ICI medications without interruption; Two patients with immune gastroenteritis and one with immune pneumonitis continued ICIs after glucocorticoid therapy and did not recur with similar iRAEs on subsequent treatment; Two patients received thyroid hormone replacement therapy after developing poor thyroid function, and continued ICI medication without weaning; Two patients developed symptoms of grade 1–2 hyperthyroidism, continued ICI treatment, rechecked thyroid function every cycle, and there was no further progression of hyperthyroidism; Two patients developed immunologic fever, but both were low‐grade fevers with the maximum temperature not exceeding 38.5°C, their body temperature dropped to the normal range after physically cooled, and the continued use of ICI medications was not affected ultimately **(**Table 3 **).**

**TABLE 3:** Adverse reactions in two groups.

	Chemoimmunotherapy group (*n* = 64)			Chemotherapy group (*n* = 65)			Total (*n* = 129)		
	All	Grade 1–2	Grade 3–4	All	Grade1–2	Grade3–4	All	Grade1–2	Grade3–4
Hematological									
Neutrophil count decreased	20 (31.25)	16 (25.00)	4 (6.25)	9 (13.85)	9 (13.85)	0	29 (22.48)	25 (19.40)	4 (3.10)
Hemoglobin count decreased	3 (4.69)	3 (4.69)	0	11 (16.92)	11 (16.92)	0	14 (10.85)	14 (10.85)	0
Platelet count decreased	14 (21.88)	8 (12.50)	6 (9.38)	9 (13.85)	5 (7.69)	4 (6.15)	23 (17.83)	13 (10.08)	10 (7.75)
Bilirubin increased	23 (35.94)	22 (34.38)	1 (1.56)	12 (18.46)	12 (18.46)	0	35 (27.13)	34 (26.36)	1 (0.78)
Transaminase increased	18 (28.13)	17 (26.56)	1 (1.56)	20 (30.77)	20 (30.77)	0	38 (29.46)	37 (28.68)	1 (0.78)
Serum albumin decreased	32 (50.00)	30 (46.88)	2 (3.13)	28 (43.08)	26 (40.00)	2 (3.08)	60 (46.51)	56 (43.41)	2 (1.55)
Non‐hematological									
Nausea or vomiting	3 (4.69)	3 (4.69)	0	5 (7.69)	5 (7.69)	0	8 (6.20)	8 (6.20)	0
Anorexia	15 (23.44)	15 (23.44)	0	11 (16.92)	10 (15.38)	1 (1.54)	26 (20.16)	25 (19.40)	1 (0.78)
Mouth ulcers	5 (7.81)	4 (6.25)	1 (1.56)	1 (1.54)	1 (1.54)	0	6 (4.65)	5 (3.88)	1 (0.78)
Diarrhea	2 (3.13)	2 (3.13)	0	4 (6.15)	4 (6.15)	0	6 (4.65)	6 (4.65)	0
Constipation	1 (1.56)	1 (1.56)	0	0	0	0	1 0.78)	1 0.78)	0
Immune capillary hyperplasia	13 (20.31)	13 (20.31)	0	0	0	0	13 (10.08)	13 (10.08)	0
Immune gastroenteritis	2 (3.13)	2 (3.13)	0	0	0	0	2 (1.55)	2 (1.55)	0
Immune hyperthyroidism	2 (3.13)	2 (3.13)	0	0	0	0	2 (1.55)	2 (1.55)	0
Immune hypothyroidism	2 (3.13)	2 (3.13)	0	0	0	0	2 (1.55)	2 (1.55)	0
Immune pneumonia	1 (1.56)	1 (1.56)	0	0	0	0	1 0.78)	1 0.78)	0
Immune fever	2 (3.13)	2 (3.13)	0	0	0	0	2 (1.55)	2 (1.55)	0
Peripheral neurotoxicity	6 (9.38)	5 (7.81)	1 (1.56)	6 (9.23)	6 (9.23)	0	12 (9.30)	11 (8.53)	1 (0.78)
Joint muscle soreness	2 (3.13)	2 (3.13)	0	1 (1.54)	1 (1.54)	0	3 (2.33)	3 (2.33)	0
Hematuria/proteinuria	3 (4.69)	3 (4.69)	0	2 (3.08)	2 (3.08)	0	5 (3.88)	5 (3.88)	0
Hand‐foot syndrome	1 (1.56)	1 (1.56)	0	2 (3.08)	2 (3.08)	0	3 (2.33)	3 (2.33)	0
Skin itch	5 (7.81)	5 (7.81)	0	3 (4.62)	3 (4.62)	0	8 (6.20)	8 (6.20)	0
Papulopustular rash	4 (6.25)	4 (6.25)	0	3 (4.62)	3 (4.62)	0	7 (5.43)	7 (5.43)	0
Allergy	3 (4.69)	3 (4.69)	0	2 (3.08)	2 (3.08)	0	5 (3.88)	5 (3.88)	0
Edema	3 (4.69)	3 (4.69)	0	1 (1.54)	1 (1.54)	0	4 (3.10)	4 (3.10)	0
Alopecia	2 (3.13)	2 (3.13)	0	1 (1.54)	1 (1.54)	0	3 (2.33)	3 (2.33)	0

## 4 DISCUSSION

In our study, the PFS of the CI group and the SC group were 9.67 months and 6.83 months, respectively, with a 2.84 months (*p* < 0.001) and the difference between the two groups was statistically significant. For example, compared with the SC group, ORRs in the CI arm increased by 22.04% (32.81% vs. 10.77%) and DCR increased by 12% (79.69% vs. 67.69%), patients with advanced BTC may benefit from the use of ICIs combined with chemotherapy as the first‐line treatment regimen. However, the study has not yet progressed to OS, the long‐term survival rate, and overall survival rate are unknown. The ORR in the CI arm of this study increased by 19.81% compared to the KEYNOTE‐028 clinical trial and 15.81% compared to the KEYNOTE‐224 clinical trial. The results indicated that ICIs plus chemotherapy are indeed more effective than a single ICI regimen,^10^, ^14^, ^15^ and the ORR in the combined treatment arm of the study also increased by 9.81% compared with the ORR after treatment with both ICIs, which is in agreement with the finding of the NCT02923934 trial.^13^ Another phase II clinical trial (NCT03486678) has been conducted by researchers to investigate the efficacy and safety of the camrelizumab plus GEMOX regimen in the first‐line treatment of patients with advanced BTC, and the primary endpoint of this clinical trial is 6 months of PFS and the secondary endpoint is ORR, the results showed that 54% (20/37) of the patients achieved objective remission and the mPFS and mOS were 6.1 months and 11.8 months respectively; the results of this study showed that compared with the GEMOX regimen without ICIs, the ORR was 14.9% higher, and camrelizumab combined with the GEMOX regimen showed improved efficacy.^16^ The finding is consistent with our retrospective study. However, in our study, the ORR in the CI arm was only 32.81%, which was significantly lower than 54% observed in this phase II clinical trial. This may result by the multiple chemotherapy regimens and ICIs included in our analysis. However, due to the small number of cases included in the study, we were unable to conduct subgroup analyses of different forms of combined chemotherapy regimens and different ICIs.

In another phase II clinical trial (NCT03875235), the efficacy and safety of the GP plus durvalumab regimen combined with or without tremelimumab in the first‐line treatment of patients with advanced BTC were compared. The results showed that the ORR in patients treated with GP plus durvalumab plus tremelimumab regimen was 50% (15/30) during the second‐line treatment, while it was 70%(33/47) during the first‐line treatment. The ORR for patients treated with GP plus durvalumab but without tremelimumab was 72% (34/47) in the first‐line treatment. It can be seen that patients who used ICIs in combination with first‐line treatment can achieve more significant improvement in ORR compared to those who only use ICIs in combination with second‐line treatment. Furthermore, using two ICIs plus chemotherapy showed no obvious benefit over only one ICI plus chemotherapy in first‐line treatment. These results indicate that ICIs plus chemotherapy could be used as a first‐line treatment strategy for patients with advanced BTC,^17^, ^18^ which is in good agreement with our current retrospective study. The phase III study (TOPAZ‐1) (NCT03875235) also found that combination chemotherapy (GP) can reduce the risk of death in patients by 20%, and one‐year survival rate can be extended from 48% to 54.1%. There were 25% patients who received combined treatment remain alive after 2 years. On contrast, the ORR for patients receiving standard chemotherapy was 10%, and could increase to 18% when treated combined with durvalumab. Moreover, both safety and tolerability were confirmed throughout the whole process, and subgroup analysis showed that Asia patients benefited more from this regimen.^17^, ^19^

In addition, in this retrospective study, we found that patients with positive PD‐L1 expression had a better prognosis on immunotherapy than patients with negative PD‐L1 expression. The PFS of patients with positive and negative PD‐L1 expression was 15.7 months and 7.4 months, respectively (*p* < 0.05). These findings are consistent with the KEYNOTE‐158 clinical trial, in which 17% of the patients with dMMR/MSI‐H achieved PR for a longer duration of time, and ICIs on advanced BTC patients with dMMR/MSI‐H showed improved responsiveness with an ORR as high as 40.9%. At present, pembrolizumab has been approved for patients with advanced BTC with MSI‐H/dMMR after failure of first‐line regimens.^2^, ^9^, ^11^, ^12^ Therefore, the therapeutic potential of immunotherapy for BTC is worthy of expectation.^20^

Several studies have shown that patients with TMB‐H generally have improved immunotherapeutic outcomes.^21^ A large clinical study covering multiple different cancers comprising 1662 patients treated with ICIs showed that, the cancer patients with higher TMB values had longer OS, Therefore, TMB‐H could be considered as a biomarker to predict the efficacy of ICIs treatments.^22^ Unfortunately, we did not found similar results in this retrospective study. In our study, the PFS of the TMB‐H group and the TMB‐L group were 8.97 and 9.01 months, respectively, with no statistically significant differences (*p* > 0.05). Some previous studies had also found that in certain tumors, patients with TMB‐H are less effective after immunotherapy than those with TMB‐L. DJ McGrail et al^25^ found that the TMB‐H ratio cannot be directly associated with the responsiveness of ICIs treatment. In cancers where CD8^+^T cells were positively correlated with neoantigen load (bladder cancer, lung cancer, and melanoma, etc), TMB‐H patients responded better to ICI than TMB‐L patients. However, in cancers where CD8^+^T cells were not correlated with neoantigen load (prostate, breast, glioma, etc), the ORR was lower in both TMB‐H and TMB‐L patients. In renal clear cell carcinoma, TMB‐H patients had even lower response rates to ICIs than TMB‐L patients.^24^ In addition, Xiaofeng Chen et al.^23^, ^25^ found that TMB‐H in patients with advanced BTC was associated with poor prognosis and poor immunotherapy effectiveness. Thus, whether TMB‐H can be used as a biomarker to predict the therapeutic effect of ICIs in patents with BTC requires further in‐depth investigation.

We found that PD‐L1 expression, CA19‐9 decline, and NLR ratio may have impacts on PFS in patients with advanced BTC. In patients with advanced BTC, the PFS of patients with decreased CA19‐9 after receiving at least 2 cycles of treatment and patients without CA19‐9 decrease was 13.3 months and 5.8 months, respectively, with a difference of 7.5 months, which was statistically significant (*p* < 0.05). Casadio et al.^26^ found the expression of certain tumor markers decreased after treatment, which was associated with patient survival and disease prognosis, but we did not find supporting evidences in the present study. NLR primarily affects immunotherapy by influencing the tumor microenvironment(TME), the PFS of patients with NLR <2.30 was 12.5 months, and the PFS of patients with NLR ≥2.30 was 7.4 months, the difference was 5.1 months which was also statistically significant (*p* = 0.002). Furthermore, multivariable regression analysis also showed that NLR is a prognostic factor affecting immunotherapy in BTC patients (*p* < 0.05). Eliza W. Beal et al.^27^ found that elevated NLR was associated with poor prognosis after radical surgical resection of the BTC. Postoperative mOS was 17.5 months longer in patients with preoperative NLR ≤5 compared to those with NLR >5 (*p* < 0.001). Thus NLR >5 had been suggested to be an independent prognostic factor for GCA syndrome. However, its correlation with prognosis for CCA survival rate was relatively poor.^27^ In addition, they found that patients with persistently high NLR at 1 month after surgery had significantly worse survival rates than those with normal or decreased NLR.^28^

In this retrospective study, we also found that patients with no previous history of tobacco and alcohol had relatively better survival compared with patients with such history. The PFS score was 8.7 versus 6.3 months (*p* < 0.05). Unfortunately, the multivariable regression analysis indicated no statistical difference (*p* > 0.05).

This retrospective study showed that TRAEs occurred in both the CI group as well as the SC group. The overall incidence of TRAEs was 70.3% and 57%, respectively. The two highest incidences of grade 3–4 hematological adverse events in both groups were thrombocytopenia in 10 (7.75%) and four (3.1%) in neutropenia patients, while grade 3–4 non‐hematologically related adverse events included only 3 cases (1 anorexia, 1 peripheral neurotoxicity, and 1 oral ulcer). All TRAEs occurred were controlled after adjusting the drug dose or after receiving symptomatic supportive care, therefore ICI application was not interrupted. In the CI group, 32.8% (21/64) of iRAEs occurred, but there was no grade 3–4 iRAEs. The most common irAEs (20.31% (13/64)) was reactive dermal capillary endothelial proliferation (RCCEP), which was grade 1–2.

Our retrospective study not only with truthfulness, but also have clinical importance and practicality. First, BTC is a kind of malignancy tumor with a low incidence, makes it difficult to carry out large‐scale clinical investigation. Second, only TOPAZ‐1 clinical trial has reached conclusions, other clinical trials are still ongoing. Thus, our retrospective study reveals that the application of immunotherapies in the first‐line treatment of advanced BTC patients could provide potential clinical guidance.

Our study also has a few limitations. First, subgroup analyses between CCA and GCA as well as between different CCA sites were not performed due to the limited number of patients included. Second, we did not conduct cancer‐related genome or exome sequencing to explore the possible association between the efficacy of immunotherapy and the genomes of patients with advanced BTC, so as to guide the clinical application of immunotherapy more accurately, and help target populations for effective screening. Third, our study has not yet reached the study endpoint of OS, the specific survival advantage of immunotherapy combined with chemotherapy for patients with advanced BTC is still unclear, the scope of application for real‐world clinical guidelines are still relatively limited. Fourth, the study incorporated three chemotherapy regimens and multiple ICIs, and did not specifically analyze the possible impact of different combination of chemotherapy regimens and ICIs on survival outcomes, nor did it explore the optimal combination of chemotherapy regimens and ICIs.

In summary, ICIs combined with chemotherapy can be recommended as first‐line treatment for patients with advanced BTC, but additional randomized controlled studies are required to determine the specific impact of such therapy on long‐term prognosis.

## AUTHOR CONTRIBUTIONS

**Zhengfeng Zhang:** Conceptualization (equal); data curation (equal); formal analysis (equal); funding acquisition (equal); investigation (lead); methodology (lead); project administration (lead); resources (lead); software (lead); supervision (equal); validation (lead); visualization (equal); writing – original draft (lead); writing – review and editing (lead). **Dazhen Wang:** Conceptualization (equal); data curation (lead); formal analysis (lead); funding acquisition (equal); investigation (equal); methodology (equal); project administration (equal); resources (equal); software (equal); supervision (lead); validation (equal); visualization (equal); writing – original draft (equal); writing – review and editing (equal). **Liu Yang:** Conceptualization (equal); data curation (equal); formal analysis (equal); funding acquisition (equal); investigation (equal); methodology (equal); project administration (equal); resources (equal); software (equal); supervision (equal); validation (equal); visualization (equal); writing – original draft (equal); writing – review and editing (equal). **Lu Zhao:** Conceptualization (equal); data curation (equal); formal analysis (equal); funding acquisition (supporting); investigation (supporting); methodology (equal); project administration (equal); resources (equal); software (equal); supervision (equal); validation (supporting); visualization (supporting); writing – original draft (supporting); writing – review and editing (supporting). **Lei Yang:** Conceptualization (supporting); data curation (supporting); formal analysis (supporting); funding acquisition (equal); investigation (equal); methodology (supporting); project administration (supporting); resources (supporting); software (supporting); supervision (supporting); validation (supporting); visualization (supporting); writing – original draft (supporting); writing – review and editing (supporting). **Jianji Zhang:** Conceptualization (supporting); data curation (equal); formal analysis (supporting); funding acquisition (equal); investigation (supporting); methodology (supporting); project administration (supporting); resources (supporting); software (supporting); supervision (equal); validation (supporting); visualization (supporting); writing – original draft (supporting); writing – review and editing (supporting). **Changjie Lou:** Conceptualization (lead); data curation (equal); formal analysis (equal); funding acquisition (lead); investigation (equal); methodology (lead); project administration (lead); resources (lead); software (equal); supervision (equal); validation (equal); visualization (equal); writing – original draft (equal); writing – review and editing (equal).

## FUNDING INFORMATION

This work is supported by a grant from the Haiyan Scientific Research Fund at Harbin Medical University Cancer Hospital (JJZD2020‐03) and Beijing Medical Award Foundation (YXJL‐2022‐0800‐0015).

## CONFLICT OF INTEREST STATEMENT

The authors declare that they have no conflict of interests with respect to this work.

## Data Availability

I confirm that my article contains a Data Availability Statement even if no data is available (list of sample statements) unless my article type does not require one. I confirm that I have included a citation for available data in my references section, unless my article type is exempt.
